# A Case of Thyrotoxicosis due to Simultaneous Occurrence of Subacute Thyroiditis and Graves' Disease

**DOI:** 10.1155/2018/3210317

**Published:** 2018-10-30

**Authors:** Kazunori Kageyama, Noriko Kinoshita, Makoto Daimon

**Affiliations:** ^1^Department of Endocrinology and Metabolism, Hirosaki University Graduate School of Medicine, 5 Zaifu-cho, Hirosaki, Aomori 036-8562, Japan; ^2^Department of Endocrinology and Metabolism, Odate Municipal General Hospital, 3-1 Yutaka-cho, Odate 017-8550, Japan

## Abstract

Subacute thyroiditis is an inflammatory disorder of the thyroid. Graves' disease is an autoimmune thyroid disease in which thyroid hormones are overproduced. Here we present a rare case of thyrotoxicosis due to the simultaneous occurrence of both diseases. Prompt diagnosis and therapy are required to prevent complications in patients with thyrotoxicosis.

## 1. Introduction

Subacute thyroiditis is an inflammatory disorder of the thyroid with characteristic presentations and clinical course. It typically presents with painful thyroid swelling and destruction-induced thyrotoxicosis. Graves' disease is an autoimmune thyroid disease, in which overproduction of thyroid hormones results in thyrotoxicosis.

Graves' disease following subacute thyroiditis is not uncommon [1], whereas Graves' disease concurrent with subacute thyroiditis is very rare, with only a few cases reported in the English literature to date [2–4]. To our knowledge, the present case is a rare report on thyrotoxicosis due to simultaneous occurrence of subacute thyroiditis and Graves' disease.

## 2. Case Report

A 75-year-old woman with cough, rhinorrhea, sore throat, and appetite loss was evaluated at our hospital for anterior cervical pain and thyrotoxicosis. The patient's height was 145 cm, and her body weight was 40.0 kg. At her first visit to our hospital, her blood pressure was 137/81 mmHg, with a regular pulse rate of 116 beats/min. Her body temperature was 37.0°C. An electrocardiogram showed sinus tachycardia. She had no family history of autoimmune thyroid diseases. Nor did she take any medicines.

**Figure 1:**
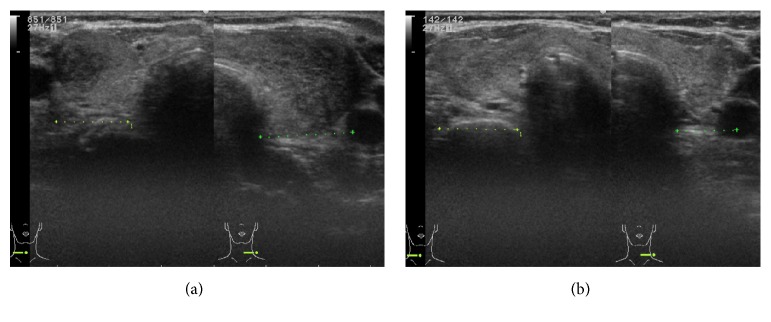
Ultrasonography of the thyroid. (a) Heterogeneous and hypoechoic areas were observed in both thyroid lobes before treatment. (b) Hypervascularity and a reduction in hypoechoic areas, indicating improvement, were observed 10 weeks after treatment initiation.

Her neck pain initially appeared on the left side and subsequently moved to the right side. Laboratory data showed a normal white cell count (8,290 cells /*µ*L) and slightly elevated C-reactive protein (3.94 mg/dL) and alkaline phosphatase (460 U/L) levels (Table 1). Thyroid hormone levels were also elevated (free triiodothyronine, 20.27 pg/mL; free thyroxine, 6.53 ng/dL; thyroglobulin, 183 ng/mL), whereas thyroid-stimulating hormone (TSH) was undetectable. Ultrasonography (US) of the thyroid revealed heterogeneous and hypoechoic areas, which are features of subacute thyroiditis, in both thyroid lobes (Figure 1). Biopsy was not performed.

**Figure 2:**
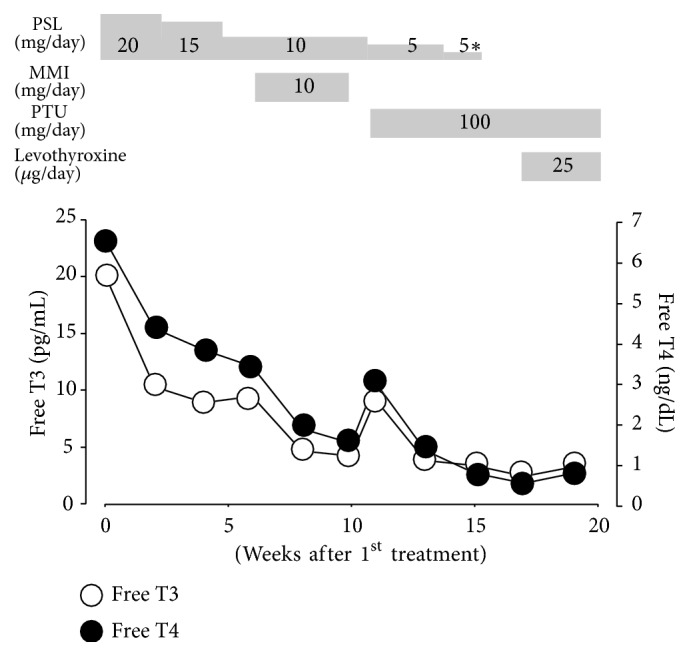
Changes in serum thyroid levels during the clinical course. 5*∗*, 5 mg every 2 days; PSL, prednisolone; MMI, methimazole; PTU, propylthiouracil; T3, triiodothyronine; T4, thyroxine.

The patient was treated with prednisolone (PSL, 20 mg/day), and her neck pain disappeared shortly thereafter. Two weeks after treatment initiation, we found strong anti-TSH receptor antibody (TRAb) and anti-thyroid-stimulating antibody positivity (Table 2). The PSL dose was gradually tapered, and thyroid hormone levels decreased, although they were still above normal 6 weeks after treatment start (Figure 2). At this time, the patient received both PSL (10 mg/day) and methimazole (MMI, 10 mg/day). Four weeks later, thyroid hormone levels improved, and US showed hypervascularity and fewer hypoechoic areas in the thyroid. However liver function worsened, and MMI treatment was therefore stopped. One week after MMI withdrawal, the damaged liver had recovered, but the thyroid hormone levels were again elevated. Propylthiouracil (PTU, 100 mg/day) was administered, and levothyroxine (25 *µ*g/day) was later added to control thyroid function.

## 3. Discussion

The patient had findings consistent with subacute thyroiditis: a painful swollen thyroid, hypoechoic lesions in the painful portion of the thyroid, mildly elevated C-reactive protein levels, elevated thyroid hormone levels, and suppressed TSH production. Moreover, her clinical course—neck pain disappearance soon after beginning treatment with PSL and gradual reduction in thyroid hormone levels—matched that of subacute thyroiditis. She also had findings consistent with Graves' disease: hypervascularity of the thyroid, persistently elevated thyroid hormone levels despite 6 weeks of PSL treatment, and reduced thyroid hormone levels following treatment with MMI and PTU. The ^99m^Tc or ^131^I thyroid uptake might be useful to distinguish Graves' disease from subacute thyroiditis, but not in all cases [3]. Taken together, simultaneous occurrence of subacute thyroiditis and Graves' disease was the cause of the thyrotoxicosis in this case.

Cases in which Graves' disease followed subacute thyroiditis have been reported [1]. Subacute thyroiditis might promote the development of autoimmune diseases by increasing the production of TRAbs and subsequent release of thyroid antigens. In support, thyroid autoantibodies have been found in patients with subacute thyroiditis [5]. On the other hand, simultaneous occurrence of subacute thyroiditis and Graves' disease is very rare because surplus IgG production requires more than 2 months [5]. The relationship between destructive thyroiditis, most likely caused by a viral infection, and Graves' disease remains unclear, although Fang et al. [4] suggest that thyroid autoimmunity precedes the development of subacute thyroiditis.

Human leukocyte antigen (HLA) subtypes have been reported to confer genetic susceptibility to autoimmune thyroid diseases [6, 7]. Genetic susceptibility has been implicated in both subacute thyroiditis and Graves' disease, with HLA-B35 linked to the former [6] and HLA-DRB1 and HLA-DQB1 to the latter [7]. Although HLA subtypes were not examined in our patient, they may have contributed to the occurrence of either or both diseases. Alternatively, the concurrence of both diseases may be coincidental.

In conclusion, we presented a case of thyrotoxicosis due to the simultaneous occurrence of subacute thyroiditis and Graves' disease. Prompt diagnosis and therapy are required to prevent complications in patients with thyrotoxicosis.

## Figures and Tables

**Table 1:** General laboratory data.

Variable	Value	Normal range
*Peripheral blood*		
White blood cells/*μ*L	8290	3500–8500
Red blood cells/*μ*L	355 × 10^4^	380–480 ×10^4^
Hemoglobin (g/dL)	10.4	11.5–15.0
Hematocrit (%)	32.2	34.0–45.0
Platelets/L	445 × 10^3^	130–350 × 10^3^

*Blood biochemistry*		
Total protein (g/dL)	6.9	6.7–8.3
Albumin (g/dL)	2.8	3.9–4.9
Aspartate aminotransferase (U/L)	34	10–35
Alanine aminotransferase (U/L)	39	7–38
*γ*-Glutamyltranspeptitase (U/L)	45	0–65
Alkaline phosphatase (U/L)	460	104–340
Creatine kinase (U/L)	41	57–236
Urea nitrogen (mg/dL)	25	8–25
Creatinine (mg/dL)	0.48	0.40–1.10
Uric acid (mg/dL)	2.5	2.3–7.0
Sodium (mmol/L)	141	137–146
Chloride (mmol/L)	104	99–110)
Potassium (mmol/L)	3.7	3.5–4.9
Calcium (mg/dL)	9.4	8.3–10.3
Plasma glucose (mg/dL)	103	70–110
Hemoglobin A1c (%)	5.8	4.6–6.2
Total cholesterol (mg/dL)	130	115–220
Triglyceride (mg/dL)	90	30–150
C-reactive protein (mg/dL)	3.94	0.00–0.30

**Table 2:** Thyroid laboratory data.

Variable	Value	Normal value
TRAb (IU/L)	16.3	0–1.9
TSAb (%)	767	0–120
Thyroglobulin (ng/mL)	183	0–33.7
TPOAb (U/mL)	9	0–15
TgAb (IU/mL)	40	0–27

TRAb, anti-thyroid-stimulating receptor antibody; TSAb, anti-thyroid-stimulating antibody; TPOAb, anti-thyroid peroxidase antibody; TgAb, anti-thyroglobulin antibody.
